# Inappropriate analysis does not reveal the ecological causes of evolution of stickleback armour: a critique of Spence et al. 2013

**DOI:** 10.1002/ece3.1179

**Published:** 2014-08-14

**Authors:** Andrew D C MacColl, Beth Aucott

**Affiliations:** School of Life Sciences, University of Nottingham, University ParkNottingham, NG7 2RD, U.K

**Keywords:** Calcium, Collinearity, Gasterosteus aculeatus, Lateral plates, Predation, Selective agents

## Abstract

In a recent paper in this journal, Spence et al. (2013) sought to identify the ecological causes of morphological evolution in three-spined sticklebacks *Gasterosteus aculeatus*, by examining phenotypic and environmental variation between populations on the island of North Uist, Scotland. However, by using simple qualitative assessments of phenotype and inappropriate measures of environmental variation, Spence et al. have come to a conclusion that is diametrically opposite to that which we have arrived at in studying the same populations. Our criticisms of their paper are threefold: (1) using a binomial qualitative measure of the variation in stickleback armour (“low” versus “minimal” (i.e., “normal” low-plated freshwater sticklebacks versus spineless and/or plateless fish)) does not represent the full range of phenotypes that can be described by quantitative measures of the individual elements of armour. (2) Their use of unspecified test kits, with a probable accuracy of 4 ppm, may not be accurate in the range of water chemistry on North Uist (1 to 30 ppm calcium). (3) Their qualitative assessment of the abundance of brown trout *Salmo trutta* as the major predator of sticklebacks does not accurately describe the variation in brown trout abundance that is revealed by catch-per-unit-effort statistics. Repeating Spence et al.’s analysis using our own measurements, we find, in direct contradiction to them, that variation in stickleback bony armour is strongly correlated with variation in trout abundance, and unrelated to variation in the concentration of calcium in the lochs in which they live. Field studies in ecology and evolution seldom address the same question in the same system at the same time, and it is salutary that in this rare instance two such studies arrived at diametrically opposite answers.

## Introduction

Our understanding of the ecological circumstances that drive adaptive evolution is poor. Where there is sufficient phenotypic diversity between local populations or closely related species, correlations between phenotype and environment may help to reveal the ecological causes of evolution (MacColl 2011). The reliability of such an approach depends partly on having good measures of phenotype and of putative environmental agents of selection.

In a recent paper, Spence et al. (2013) sought to identify the ecological causes of morphological evolution in three-spined sticklebacks *Gasterosteus aculeatus*, by examining phenotypic and environmental variation between populations on the island of North Uist, Scotland. Three-spined sticklebacks (hereafter “sticklebacks”) provide an excellent model for such work because isolated populations in freshwater evolve rapidly in response to local conditions (Jones et al. 2012; MacColl et al. 2013; Reimchen et al. 2013). Populations of sticklebacks on North Uist are peculiarly suitable for this type of study because the numerous freshwater lochs on the island are isolated to a large degree, species poor, and relatively simple, while encompassing great variation in biotic and abiotic variables on small spatial scales (MacColl et al. 2013).

Spence et al. (2013) used the natural variation on North Uist to examine the correlations between the extent of development of external bony armour, calcium concentration, and the abundance of an important predator, the brown trout *Salmo trutta*. Their paper was a follow-up to the work of Giles (1983) who first proposed that the evolution of bony armour in sticklebacks might be driven by calcium availability, after establishing a correlation between the two for North Uist lochs. The conclusion of Spence et al. (2013) supported that of Giles, in finding a correlation between armour and calcium, but no correlation between armour and trout abundance. However, by using simple qualitative assessments of phenotype and inappropriate measures of environmental variation, Spence et al. have come to a conclusion that is diametrically opposite to that which we have arrived at in studying the same populations. Our criticisms of their paper are threefold: (1) using a binomial qualitative measure of the variation in stickleback armour (“low” versus “minimal” (i.e., “normal” low-plated freshwater sticklebacks versus spineless and/or plateless fish)) does not represent the full range of phenotypes that can be described by quantitative measures of the individual elements of armour. (2) Their use of an unspecified LaMotte test kit, with a probable accuracy of 4 ppm, may not be sufficiently accurate in the range of water chemistry on North Uist (1 to 30 ppm calcium). (3) Their qualitative assessment of the abundance of brown trout *Salmo trutta* as the major predator of sticklebacks does not accurately describe the variation in brown trout abundance that is revealed by catch-per-unit-effort statistics.

## Methods

### Stickleback sampling

We studied many of the same populations as Spence et al. (2013), between 2007 and 2013. Samples of sticklebacks were collected during the breeding season (April and May) in 2008, 2010 and 2011, using minnow traps (Gee’s, Dynamic Aqua, Vancouver, Canada) set in 30 cm to 3 m of water for 1 to 3 nights (normally 2). Traps were emptied into buckets, and samples of fish taken haphazardly from these buckets were euthanized with an overdose of MS222. Fish were preserved in 70% ethanol and returned to the laboratory in Nottingham. Examination of the otoliths of our breeding season samples shows that none of the fish were young of the year (MacColl et al. 2013). Approximately 10 fish from each population, spanning the range of standard lengths collected, were selected for staining. Fish were transferred to formalin for 2 weeks, stained with 0.04% alizarin red in 1% KOH for 24 h, rinsed in tap water for 24 h, and stored in propanol. Fish were photographed individually with a Nikon D60 digital camera and 60 mm macro Nikkor lens. The following attributes of the bony armour of each fish were recorded from the photographs: length of the first and second dorsal spines, length of the left pelvic spine, length of the pelvic girdle, height of the pelvic vertical process, height of the largest thoracic plate and the total number of lateral plates on the left side.

### Environmental measures

A filtered water sample, acidified with nitric acid, was collected in May 2011 from each of 21 of the North Uist freshwater lochs analyzed by Spence et al. These samples were returned to the University of Nottingham for analysis of calcium concentrations by quadrupole inductively coupled plasma mass spectrometry (ICP-MS, Thermo-Fisher XSeries^II^, Waltham, MA, USA). Calcium concentrations were measured in collision cell with kinetic energy discrimination (CCT-KED) mode with a dwell time of 10 msec and maximum settle time of 15,000 μ-sec with 500 sweeps per element. Calibration standards in the range 0–30 ppm were used with scandium, germanium, rhodium, and iridium internal standards.

The local North Uist Angling Club has kept a record of all brown trout landed during club fly-fishing competitions from 1956 onwards, as well as the number of anglers taking part and the number of hours for which they fished. We used their data from 1956 to 2006 to estimate the mean number of trout landed per angler per hour (“trout catch rate” is a measure of catch-per-unit-effort) and the mean mass of captured brown trout (“mean trout mass”, all trout caught were weighed) in 17 of the lochs analyzed by Spence et al. We are confident that these data provide good indices of the brown trout in these lochs (MacColl et al. 2013), given the standardized method of capture (only fly-fishing), generally large sample sizes, large differences in density and size between lochs (see “Results”), the length of time over which the data were collected, and the fact that the data are internally consistent (see “Results”).

### Statistical analysis

All statistical analyses were performed in Genstat (15th edition). Principal Components Analysis (PCA) (using a correlation matrix approach) was used to summarize the measurements of ectodermal bony “armour”. Each armour component (except number of lateral plates, which is independent of size in our data set) was first size-standardized, by taking residuals from a regression of each trait on body size (standard length). We used simple (Pearson’s) correlations to quantify the associations between our measures of environmental variation and those of Spence et al. The relationships between trout catch rate and mean trout mass and between armour and calcium and mean trout size were investigated with generalized linear models (GLMs) with normal errors and identity link functions.

## Results

### Our data

The lochs that we sampled overlap substantially, but not completely, with those sampled by Spence et al. (2013). We have calcium measurements from 21 of their lochs, trout data from 17 and armour data from 16. The armour data comprise samples representing 12 of the 19 catchments (in which lochs are often connected) sampled by Spence et al.

**Figure 1:**
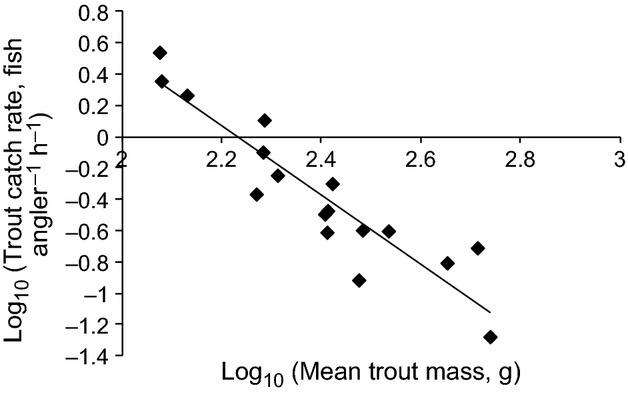
The relationship between brown trout catch rate and mean brown trout mass for 17 freshwater lochs on the island of North Uist, Scotland.

Our measurements of calcium concentrations for 21 lochs in the Spence et al. (2013) paper varied between 1.42 and 31.5 mg L^−1^. Trout catch rates varied between 0.04 and 3.5 trout per angler per hour (i.e., between very low and very high density), and mean trout mass varied between 119 and 548 g (i.e., between small and large average size). Trout catch rate varied with mean trout mass according to a power relationship, with (log) mean trout mass explaining 84% of the variation in (log) trout catch rate (*F*_1,15_ = 83.0, *P* < 0.001, Fig. 1), such that where trout are common they are also small.

The first (correlation matrix) principal component of the seven measurements of ectodermal bone accounted for 70% (i.e., first eigenvalue = 4.90) of the variation. All measurements had positive loadings on this principal component (Table 1), suggesting that it is a good measure of overall ectodermal boniness and we term it “armour PC”.

**Table 1:** The loadings of component measures of ectodermal bone on the first (“armour PC”) principal component. All component measures included in the PCA were residuals from the simple regression of the trait on body size (standard length), except number of lateral plates

Trait	PC1 loading
First dorsal spine	0.371
Second dorsal spine	0.418
Left pelvic spine	0.407
Height of pelvic process	0.414
Length of pelvis	0.389
Number of lateral plates	0.226
Lateral plate height	0.385

### Relationships between our data and those of Spence et al

**Figure 2:**
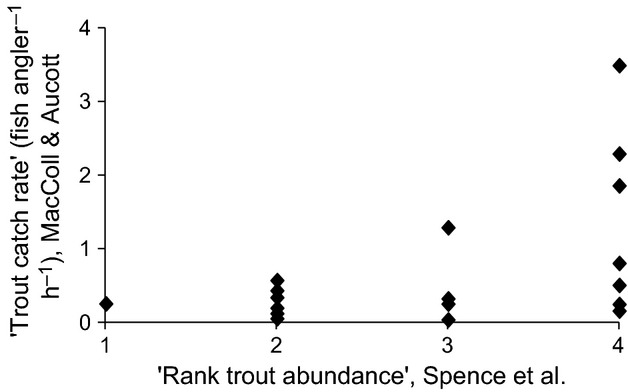
The relationship between brown trout catch rate and rank trout abundance, as used by Spence et al. 2013, for 17 freshwater lochs on the island of North Uist, Scotland.

Figure 2 shows the relationship between trout catch rate and Spence et al. (2013)’s “rank trout abundance”. The correlation between the two is significant (*r* = 0.498, *P* = 0.036), but the relationship is noisy, especially at higher values of rank trout abundance, suggesting that both are not good measures of trout abundance.

**Figure 3:**
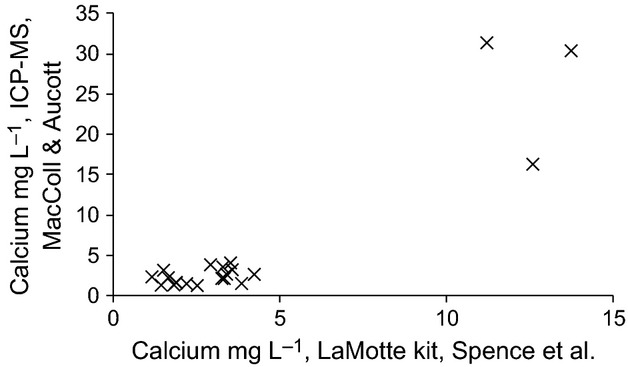
The relationship between calcium concentration, measured by inductively coupled plasma mass spectrometry and by a kit, as used by Spence et al. 2013, for 21 freshwater lochs on the island of North Uist, Scotland.

Figure 3 shows the relationship between calcium measured by ICP-MS and the LaMotte kit used by Spence et al. (2013). There is a strong correlation (0.922, *P* < 0.001) between the two across the whole range of the data, but this is driven by the two widely spaced clusters of points for machair and acid lochs. Within the low calcium (<5 mg·L^−1^), acid loch cluster, the relationship between the two is poor (*r* = 0.396, *P* = 0.104).

**Figure 4:**
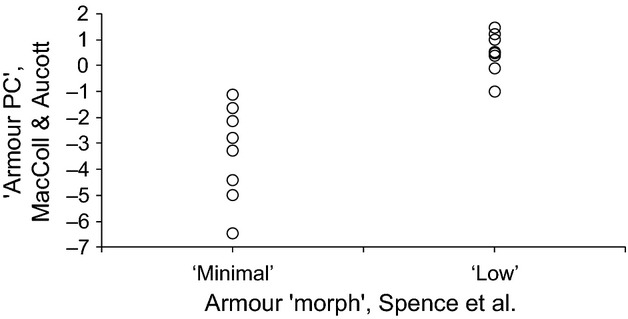
The relationship between the first principal component “armour PC” of seven measures of ectodermal bone and the armour “morph” classification, as used by Spence et al. 2013, for 16 freshwater lochs on the island of North Uist, Scotland.

Figure 4 shows the distribution of values of the armour first principal component (armour PC), in relation to the binomial “minimal” versus “low” binomial classification used by Spence et al. The two approaches are consistent, in that there is no overlap in the distributions of armour pc measures between minimal and low-plated fish, but the principal component approach reveals substantial variation in armour within “morphs” beyond that revealed by qualitative classification.

**Figure 5:**
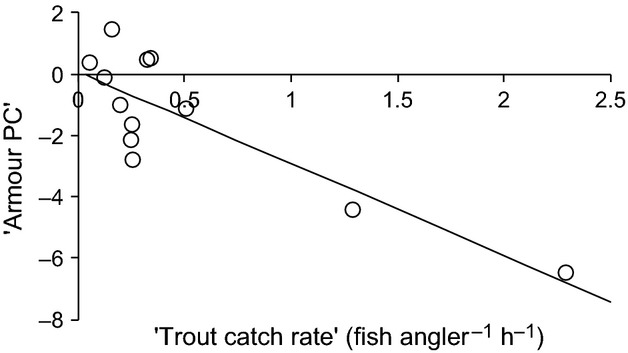
The relationship between stickleback armour and trout catch rate, for 16 freshwater lochs on the island of North Uist, Scotland.

There were 12 populations in our dataset which all had measures of “armour PC” (PC1 of bone measurements), calcium (ICP-MS) and trout catch rate (fly fishing catch-per-unit-effort). In a generalized linear model, using armour PC as the dependent variable and our measures of calcium and trout catch rate and their interaction as the explanatory variables, only trout catch rate was significant (Table 2, Fig. 5), explaining 69% of the variation in armour. The outcome of this analysis is unchanged when a further nine lochs, not studied by Spence et al., are added. We also analyzed the relationship between Spence et al.’s binomial armour “morph” classification and our measures of the environmental variables, in a GLM with binomial errors and a logit link function. Trout catch rate was again the only significant explanatory variable (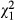 = 6.96, *P* = 0.008, calcium: 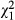 = 2.43, *P* = 0.12, trout catch rate × calcium: 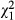 = 0.06, *P* = 0.81). These analyses suggest that sticklebacks lose their armour where trout are common (and small).

**Table 2:** The results of a GLM to investigate the relationship between stickleback armour (“armour PC”) and trout catch rate, calcium and their interaction, for 12 freshwater lochs on the island of North Uist, Scotland. The model had normal errors and an identity link function. The final model explained 69.4% of the variation in armour PC

Explanatory variable	Wald *F*	df	*P*
Trout catch rate (fish angler-1 h^−1^)	26.00	1, 10	<0.001
Calcium mg L^−1^	1.68	1, 9	0.23
Trout catch rate × calcium	0.31	1, 8	0.59

## Discussion

We believe that our use of quantitative approaches to measure the variation in trout abundance and extent of the armour phenotype is more robust and more informative than the qualitative approaches adopted by Spence et al. (2013). Coupled with the use of ICP-MS rather than kits to measure calcium concentrations, we are confident that our finding that there is a relationship between stickleback armour and trout abundance, but not calcium, is the correct one. We therefore concur with Reimchen et al. (2013) that predation is a more important selective agent shaping armour than is calcium concentration.

Measurement of the components of stickleback armour (spines, plates and pelvis) (Moodie and Reimchen 1976) reveals substantial variation within morph classes that allows more complete description of phenotypic variation, and increases the power to detect relationships with environmental variables (Reimchen et al. 2013). However, had we used Spence et al. (2013)’s morph classification system, we would still have arrived at our conclusion, which suggests that the main problem in Spence et al.’s analysis resides in their measurement of environmental variables.

The strong power relationship in our angling club dataset, with higher trout density associated with smaller trout size, is exactly what is expected from macroecological considerations (e.g., Damuth 1981; Dunham and Vinyard 1997; Cohen et al. 2012), and shows that our data are internally consistent. Any measure of animal abundance is subject to error, and our trout catch rate data are unlikely to be any different. However, the use of a large dataset gathered over many years and the fact that it is internally consistent give us some confidence that “trout catch rate” does represent a meaningful measure of trout density. It is interesting and important to note that the relationship between armour and trout catch rate in the North Uist lochs is opposite to that which, naively, might be expected *a priori*: sticklebacks are *less* armoured where trout are more common. There are two possible explanations for this, which are not mutually exclusive. Firstly, trout are bigger where they are less abundant (Fig. 1) and it may only be large trout that consume sticklebacks. Secondly, resource conditions are likely poorer in those lochs where trout are smaller, and this may make it very expensive for sticklebacks to grow armour (which they may not need) (MacColl et al. 2013). Preliminary analyses of trout stomach contents are certainly consistent with the first of these two hypotheses.

The poor agreement between rank trout abundance and trout catch rate calls into question Spence et al. (2013)’s analysis of the association between stickleback behaviour and predation risk. Although we do not have trout catch rate data for all of the populations that they used in their trials, the mean trout catch rate for three of the low morph populations which they describe as having high brown trout abundance (0.17 ± 0.08 fish angler^−1^ h^−1^) was lower than the average of seven populations (0.28 ± 0.07 fish angler^−1^ h^−1^, two minimal and five low morph) described as having low brown trout abundance. Taken at face value and contrary to Spence et al.’s conclusion, the data are consistent with low morph fish, which tend to occur with larger brown trout, being more nervous.

Inductively coupled plasma mass spectrometry provides the standard way to measure metal ions at low concentrations, and is generally accepted to be more accurate than the use of kits. This may be especially true at the very low calcium concentrations in North Uist freshwaters.

It is unusual in ecological and evolutionary studies that different groups are addressing the same questions in the same species in the same locations at the same time. It is then salutary that when it does occur the conclusions reached can be diametrically opposite. In fact, this system is typified by strong collinearity of environmental variables across lochs, which is common in ecological systems (Graham 2003), and makes difficult any attempt to attribute cause of evolved differences to any individual ecological factor (MacColl 2011, 2012). However, we believe that causation ultimately can be established by careful quantification and suitable experiments, and that the factors involved may turn out to be surprising. when ecological variation is comprehensively quantified.

## References

[b1] Cohen JE, Plank MJ, Law R (2012). Taylor’s law and body size in exploited marine ecosystems. Ecol. Evol.

[b2] Damuth J (1981). Population density and body size in mammals. Nature.

[b3] Dunham JB, Vinyard GL (1997). Relationships between body mass, population density, and the self-thinning rule in stream-living salmonids. Can. J. Fish. Aquat. Sci.

[b4] Giles N (1983). The possible role of environmental calcium levels during the evolution of phenotypic diversity in Outer Hebridean populations of the three-spined stickleback, *Gasterosteus aculeatus*. J. Zool.

[b5] Graham MH (2003). Confronting multicollinearity in ecological multiple regression. Ecology.

[b6] Jones FC, Grabherr MG, Chan YF, Kingsley DM (2012). The genomic basis of adaptive evolution in threespine sticklebacks. Nature.

[b7] MacColl ADC (2011). The ecological causes of evolution. Trends Ecol. Evol.

[b8] MacColl ADC (2012). The story of O: reply to Moya-Larano. Trends Ecol. Evol.

[b9] MacColl ADC, El Nagar A, de Roij J (2013). The evolutionary ecology of dwarfism in three-spined sticklebacks. J. Anim. Ecol.

[b10] Moodie GEE, Reimchen TE (1976). Phenetic variation and habitat differences in Gasterosteus populations of the Queen Charlotte islands. Syst. Zool.

[b11] Reimchen TE, Bergstrom C, Nosil P (2013). Natural selection and the adaptive radiation of Haida Gwaii stickleback. Evol. Ecol. Res.

[b12] Spence R, Wootton RJ, Barber I, Przybylski M, Smith C (2013). Ecological causes of morphological evolution in the three-spined stickleback. Ecol. Evol.

